# P-981. Revalidation and Calibration of a Risk Scoring Tool to Optimize Time to Effective Therapy in Multidrug-Resistant Pseudomonas

**DOI:** 10.1093/ofid/ofaf695.1180

**Published:** 2026-01-11

**Authors:** Nhu N Q Le, Hyunuk Seung, Megan E Dunning, Emily L Heil, Kimberly C Claeys

**Affiliations:** University of Maryland School of Medicine, Baltimore, MD; University of Maryland School of Pharmacy, Baltimore, MD; University of Maryland, Baltimore, Maryland; University of Maryland School of Pharmacy, Baltimore, MD; University of Maryland Baltimore, Baltimore, Maryland

## Abstract

**Background:**

Multidrug-resistant *Pseudomonas aeruginosa* (MDR PSA) is an exigent threat due to antibiotic resistance, lack of suitable rapid diagnostics, and delays in initiating active therapy. Locally, Zou et al. (2023) built a simplified risk scoring tool to predict MDR PSA and guide empiric antibiotic therapy. Our goal was to assess the accuracy and calibration of this tool in an updated patient population using the Infectious Diseases Society of America’s standardized definition of difficult-to-treat (DTR) PSA.

**Methods:**

Retrospective cohort study of adult ICU patients with PSA-positive blood or respiratory cultures from 2021-2024. The developed Zou et al. (2023) risk scoring tool included: previous MDR PSA within 6 months of admission, not present on admission (POA), ongoing hemodialysis, and > 4 anti-pseudomonal antibiotics within 30 days of isolation. For model development, dataset was divided into a training cohort and a test cohort. Further, beta coefficients were estimated using Firth’s method and transformed into a risk score by normalizing coefficients. The logistic regression models were trained using different sets of predictors from above.

**Results:**

Among 197 patients, 27 (14%) had MDR PSA and 11 (6%) had DTR PSA. DTR PSA patients were more likely than non-DTR PSA patients to have previous MDR PSA (9% vs 5%, p = 0.4), not have infection POA (9% vs 26%, p = 0.3), hemodialysis (64% vs 28%, p = 0.08), and > 4 previous anti-pseudomonal antibiotics (72% vs 35%, p = 0.02). Three models were developed with combinations of different risk factors. All had low sensitivity (18%), but Model 2 has the highest accuracy (c-statistics = 0.7, 95% CI: 0.6–0.9) while Model 3 had highest specificity (96%). Model 3 correctly classifies the highest true positive and negative cases (91.9%), followed by Model 2 (91.4%). Calibration curves (Figure 1) were created for all models and Model 2 had the best overall alignment between predicted and observed event rates.

**Conclusion:**

Model 2 was the best model, especially to screen high-risk patients for urgent targeted antibiotic therapy with minimal false positives. However, additional analysis is required before clinical implementation. Validation with larger patient datasets that contain more positive cases is needed to develop a higher sensitive model.

**Disclosures:**

Emily L. Heil, PharmD, MS, Wolters Kluwer: Advisor/Consultant Kimberly C. Claeys, PharmD, PhD, bioMérieux: Advisor/Consultant|bioMérieux: Honoraria

